# Surveillance of Tick‐Borne Encephalitis Virus—Comparison of Vaccination‐ and Infection‐Induced Seroprevalences in Lower Bavaria, Germany

**DOI:** 10.1002/jmv.70514

**Published:** 2025-07-24

**Authors:** Philipp Girl, Anne Schindler, Silke Martin, Gerhard Dobler, Johannes P. Borde

**Affiliations:** ^1^ Department of Veterinary Sciences Bacteriology and Mycology, Institute for Infectious Diseases and Zoonoses, Faculty of Veterinary Medicine LMU Munich Munich Germany; ^2^ Praxis Prof. Borde & Kollegen Oberkirch Germany; ^3^ Bavarian Red Cross Blood Donation Service (BSD) Munich Germany; ^4^ Bundeswehr Institute of Microbiology Munich Germany; ^5^ German Centre for Infection Research (DZIF) Partner Site Munich Munich Germany; ^6^ Department of Infectious Diseases and Tropical Medicine LMU Center of Medicine Munich Germany; ^7^ Department of Parasitology University of Hohenheim Stuttgart Germany; ^8^ Department of Medicine II University Medical Centre Freiburg, Division of Infectious Diseases, Faculty of Medicine, University of Freiburg Freiburg im Breisgau Germany

**Keywords:** epidemiology, NS1, serology, seroprevalence, TBEV

## Abstract

Tick‐borne encephalitis virus (TBEV) is an emerging flavivirus in Europe and Asia, causing severe neurological disease in humans. Recent advances in serological diagnostics, in particular the detection of antibodies against the nonstructural protein 1 (NS1), enable epidemiological research by differentiation of vaccine‐ and infection‐induced antibodies. This assay facilitates precise studies on TBEV seroprevalence. Our study investigates the TBEV seroprevalence in the district of Passau and the surrounding high‐risk areas in Lower Bavaria, Germany. A total of 1339 serum samples from voluntary blood donors were examined using a combination of IgG ELISA, NS1‐antibody ELISA, and micro‐neutralization test (NT). The results showed a TBEV‐specific IgG antibody prevalence of 84.8% (1135/1339). Of these, 3.6% (41/1135) were NS1‐antibody positive samples, which indicates infection‐related antibodies. The vaccination coverage rate was estimated at 81.2% (1094/1339), which is in line with the results from neighboring Austria but exceeds previous vaccination estimates for Bavaria. The study showed a discrepancy between the officially reported incidences (3.7 new cases/100 000 inhabitants annually) and the actual infection rate based on NS1‐antibody seroprevalence (180.6 infections/100 000 inhabitants annually). The manifestation index was calculated at 2.1%, confirming that the majority of TBE infections are subclinical or asymptomatic. The results emphasize the usefulness of NS1‐antibody‐based diagnostics in the accurate assessment of TBEV prevalence and highlight the need for improved surveillance in endemic areas.

## 1 Introduction

Tick‐borne encephalitis virus (TBEV) is an emerging tick‐borne viral pathogen that causes severe, sometimes life‐threatening infections involving the central nervous system (CNS) in humans [1]. It is the most crucial tick‐borne flavivirus in Europe and Asia, with an estimated 10 000–15 000 human cases reported annually [2]. TBEV belongs to the Flaviviridae family and the *Flavivirus* genus, which also includes other important pathogens such as yellow fever virus (YFV), Japanese encephalitis virus (JEV), dengue virus (DENV), Zika virus (ZIKV), and West Nile virus (WNV). Three genetic TBEV subtypes had been established: Western (TBEV‐EU), Far Eastern (TBEV‐FE), and Siberian (TBEV‐Sib) [3]. Recent phylogenetic studies propose at least two further subtypes, the Baikalyan and Himalayan [3]. In Central Europe, however, only the European subtype of TBEV has been detected so far [4]. The geographical distribution of TBEV is vast, spanning from Japan in the East to France and the British Isles in the West, with the virus steadily expanding its range into more northern regions, including parts of Russia, Sweden, and Finland [5]. The occurrence of TBEV is strictly linked to the presence of vector‐competent ticks, with *Ixodes ricinus* being the predominant vector in Europe, while *Ixodes persulcatus* plays a similar role in the Far East and Asia [6]. Humans are accidental hosts, contracting the virus mainly through tick bites, although in rare cases, infection can occur through the consumption of untreated milk or milk products from infected ungulate [7]. In Germany, as in many other European Countries, TBE has been a notifiable disease since 2001. Since then, more than 9100 cases of TBE have been reported in Germany [8]. The clinical manifestation of TBE in humans is often biphasic. The initial phase is characterized by nonspecific flu‐like symptoms, which may be followed by an asymptomatic period [6]. In about one‐third of cases, the disease progresses to a second phase characterized by high fever and severe neurological symptoms, including headaches, photophobia, vomiting, tremors, impaired consciousness, cognitive deficits, paralysis, and, in rare cases, even death [9]. Direct detection of TBEV by isolation or by PCR is usually only successful in blood samples taken during the viremic phase of the disease at the very beginning [9]. However, most patients present to healthcare providers in the second (neurological) phase of the disease, when TBEV infection can only be diagnosed serologically by detecting specific antibodies [9].

The only effective method of preventing the disease is the TBE vaccination; once the basic immunization schedule has been completed, the available TBE vaccines achieve an overall efficacy of 95% [10]. For residents and travelers in areas at risk, vaccination is therefore strongly recommended by national and international public health organizations, in Germany by the Standing Committee on Vaccination (STIKO) at the Robert Koch Institute (RKI) [10, 11, 12]. Nevertheless, the vaccination coverage rate in Germany is only moderate and varies between 10% and 50% in high‐risk areas [13]. Serological studies carried out in the 1980s indicate that 70%–95% of TBEV infections in humans are either subclinical or asymptomatic [2]. Since the introduction of TBE vaccines, seroepidemiological studies have been difficult to conduct in the last 40 years, as differentiation between vaccine‐induced and infection‐induced antibodies against TBEV was not possible. In addition, the occurrence of cross‐reacting flaviviruses such as WNV, Usutu virus (USUV), DENV, and ZIKV in Central Europe as well as travel‐associated vaccinations against YFV or JEV can complicate seroepidemiological investigations [14]. Recently, a new testing approach based on the detection of antibodies against the nonstructural protein 1 (NS1) of TBEV has been successfully introduced [15, 16]. The NS1 protein is produced during flavivirus infection in the cells during virus replication and released into the bloodstream, which induces the production of antibodies in the infected person [16]. However, the available inactivated vaccines do not contain NS1, in conclusion, the detection of antibodies against this protein is a reliable indicator of a current and past TBEV infection [17]. This technology can therefore be used to differentiate between infection‐ and vaccine‐related antibodies. The use of TBEV NS1‐based antibody detection, which enables a reliable distinction between infection‐ and vaccine‐induced antibodies, has opened the door for true seroprevalence studies in the context of TBE vaccination. With the first diagnostic ELISA based on this principle now validated according to DIN EN ISO 15189, it is for the first time possible to accurately determine infection rates without interference from vaccine‐induced antibodies—something that was previously only achievable through indirect estimations. In a recently published study, the new NS1‐antibody ELISA was used for the first time in a seroprevalence study in a high‐endemic area in Baden‐Wurttemberg [18]. The relevance of TBEV infections and corresponding seroprevalence studies was demonstrated here: a 7‐fold increase in TBE seroprevalence of natural TBEV infections has been shown over the last 40 years compared to studies before the introduction of vaccines. Just as alarming was the fact, that the actual incidence of the disease, depending on the district, was 50–100 times higher than the incidence assumed by the RKI, which is estimated exclusively on reported human case numbers.

In this study, we present seroprevalence data from the district of Passau and surrounding districts, a TBE high‐risk area in Lower Bavaria, Germany, and districts with the highest vaccination rate in Germany (RKI). As in the study by Euringer et al. [18], a combination of tests was used to determine the overall seroprevalence of TBEV, break it down into vaccine‐ and infection‐induced antibodies, and determine the manifestation index by comparing the results with the reporting data from the local public health authorities. In addition, the study in a second high‐risk area should provide information on whether a similarly striking gap between the assumed incidence based on the number of notified cases in humans and the actual incidence, as demonstrated for Baden‐Württemberg, can also be proven here.

## 2 Material and Methods

### 2.1 Samples

The study used residual human serum samples, collected in collaboration with the Bavarian Red Cross Blood Donation Service (Munich, Germany). For each sample, there is an anonymized data set including the parameters age, gender, and zip code was collected. The samples were collected between March and September 2024 in Passau, Lower Bavaria, Germany. After collection, each sample was centrifuged at 3000 rpm for 10 min and then stored at −20°C until further analysis.

### 2.2 Ethical Statement

A formal approval of an ethics committee was not required, because each donor gave, on a routine basis, written consent to the potential scientific use of leftover samples in anonymized form.

### 2.3 Definitions of Tick‐Borne Encephalitis Cases, Manifestation Index, and Incidence

The case definitions used in this study are published by the RKI. The German case definition differs from the definition of the European Center for Disease Prevention and Control (ECDC) as it also includes febrile forms of TBEV infections without CNS symptoms. Since 2001, the RKI has made the reported TBEV infections in humans freely available in various spatial and temporal resolutions [19].

The average annual incidence of notified TBE cases, the average annual NS1‐antibody‐based incidence, and the manifestation index were calculated as described by Euringer et al. [18]. The reported TBE incidences for the analyzed regions, as well as the TBE case numbers for the calculation of the manifestation index, were obtained from the German Ministry of Health via the service portal of the RKI (see Table 1) [8]. According to current knowledge, an average detectability of NS1‐specific IgG antibodies of about 20 years can be assumed (data not shown [15]). The incidence data, averaged over a period of 20 years, were retrieved accordingly.

**Table 1:** Details on the distribution of donors among the districts sampled, including the respective reported incidence of the districts.

County	Samples [*n*]	Proportion [%]	Reported annual mean TBE incidence [2005–2024/100 000 inhabitants]	Reported number of inhabitants
Cham	1	0.1	3.8	130 964
Deggendorf	17	1.3	2.0	123 129
Dingolfing‐Landau	1	0.1	1.0	101 477
**Freyung‐Grafenau**	**247**	**18.4**	**6.6**	**79 603**
Hof	2	0.1	1.9	92 675
Kelheim	3	0.2	1.2	125 845
Landshut	2	0.1	1.3	161 631
**Passau**	**983**	**73.4**	**3.2**	**197 863**
Regen	1	0.1	4.3	77 940
Regensburg	45	3.4	2.1	200 264
Rosenheim	3	0.2	1.7	256 815
Rottal‐Inn	19	1.4	2.8	120 475
Stadt Passau	10	0.7	3.5	51 907
Straubing‐Bogen	2	0.1	1.8	104 086
Traunstein	3	0.2	3.9	172 021
Total	1339	100		

*Note:* The districts Freyung‐Grafenau and Passau are highlighted in bold as they represented the primary recruitment areas, together accounting for the vast majority of participants (91.4%), with 983 (73.4%) and 247 (18.4%) blood donors, respectively.

### 2.4 Screening Enzyme‐Linked Immunosorbent Assays (ELISAs)

**Figure 1:**
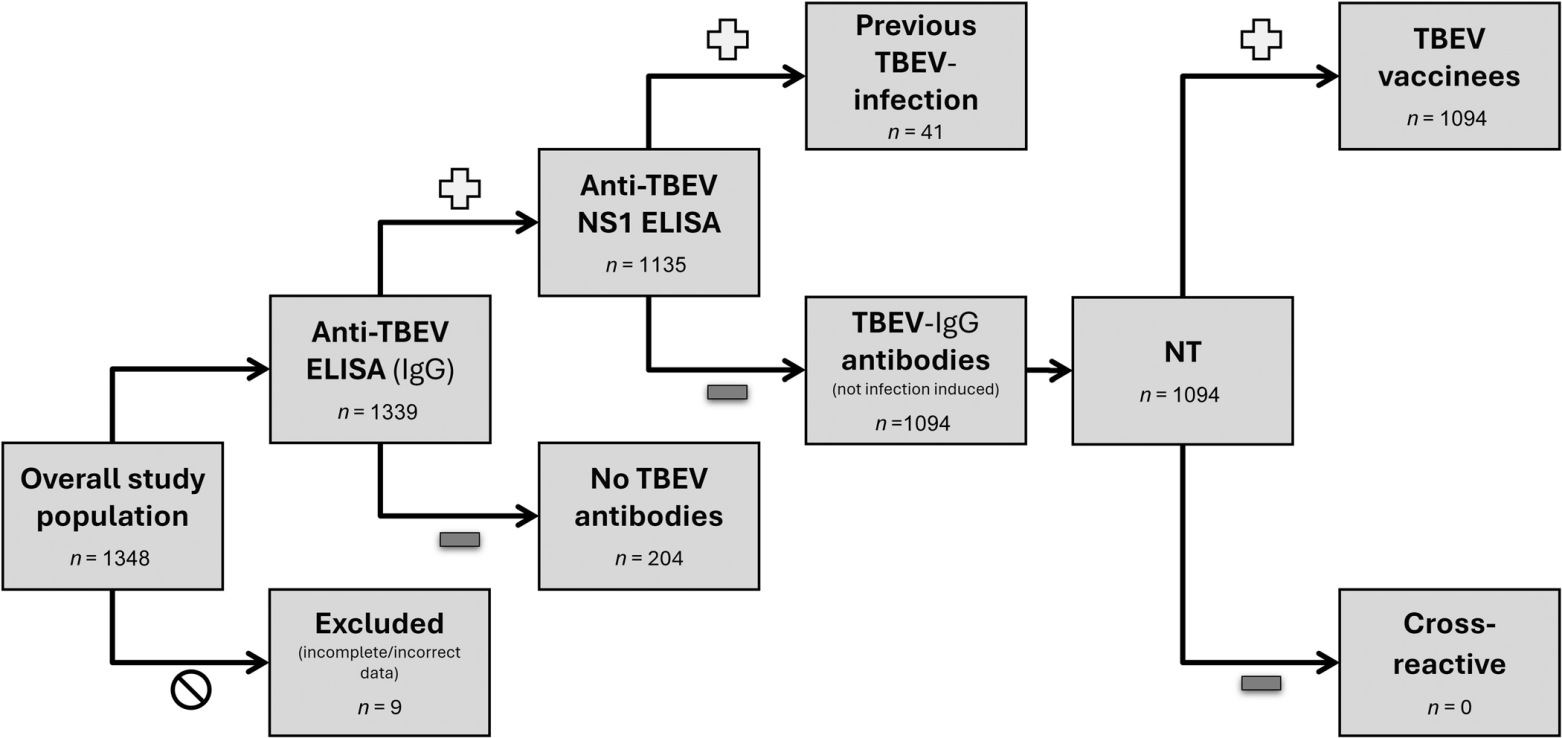
Overview of the sample flow algorithm used, and the tests performed in the individual sections.

For the initial screening of blood samples for TBEV‐specific IgG antibodies, a commercial TBEV ELISA (Euroimmun AG, Lübeck, Germany) was used and applied according to the manufacturer's instructions. All samples that tested positive or borderline in the screening test were subsequently tested with an in‐house TBEV NS1 IgG ELISA, as described previously [15]. Sera with a borderline result in the NS1‐antibody ELISA were retested and, if the result was again borderline or positive, assessed as positive, or, if the result was negative in the repeat test, assessed as negative. Figure 1 shows an overview of the sample flow algorithm used in the study and the tests used in the individual sections.

### 2.5 Micro‐Neutralization Test (NT)

All sera that gave a positive or borderline result in the screening ELISA but tested negative in the NS1‐antibody ELISA were additionally tested in a neutralization test to exclude false‐positive results in the screening test due to cross‐reactions with other flavivirus vaccination or infection. The neutralization test was performed as a NT, adapted to TBEV, according to the standard procedure as previously described [20, 21]. The antibody titer corresponded to the highest serum dilution showing complete inhibition of cytopathic effect (CPE) in both wells were reported. Thus, the samples were classified as either “NT negative” (titer < 1:10), NT borderline (titer = 10) or “NT positive” (titer ≥ 1:20). Sera with a borderline result in the NT were retested and, if the result was again borderline or positive, assessed as positive, or, if the result was negative in the repeat test, assessed as negative.

### 2.6 Statistical Analysis

All statistical analyses were performed using IBM SPSS Statistics 30 for Windows (IBM Corp., Armonk, NY, USA).

## 3 Results

### 3.1 Study Population

**Figure 2:**
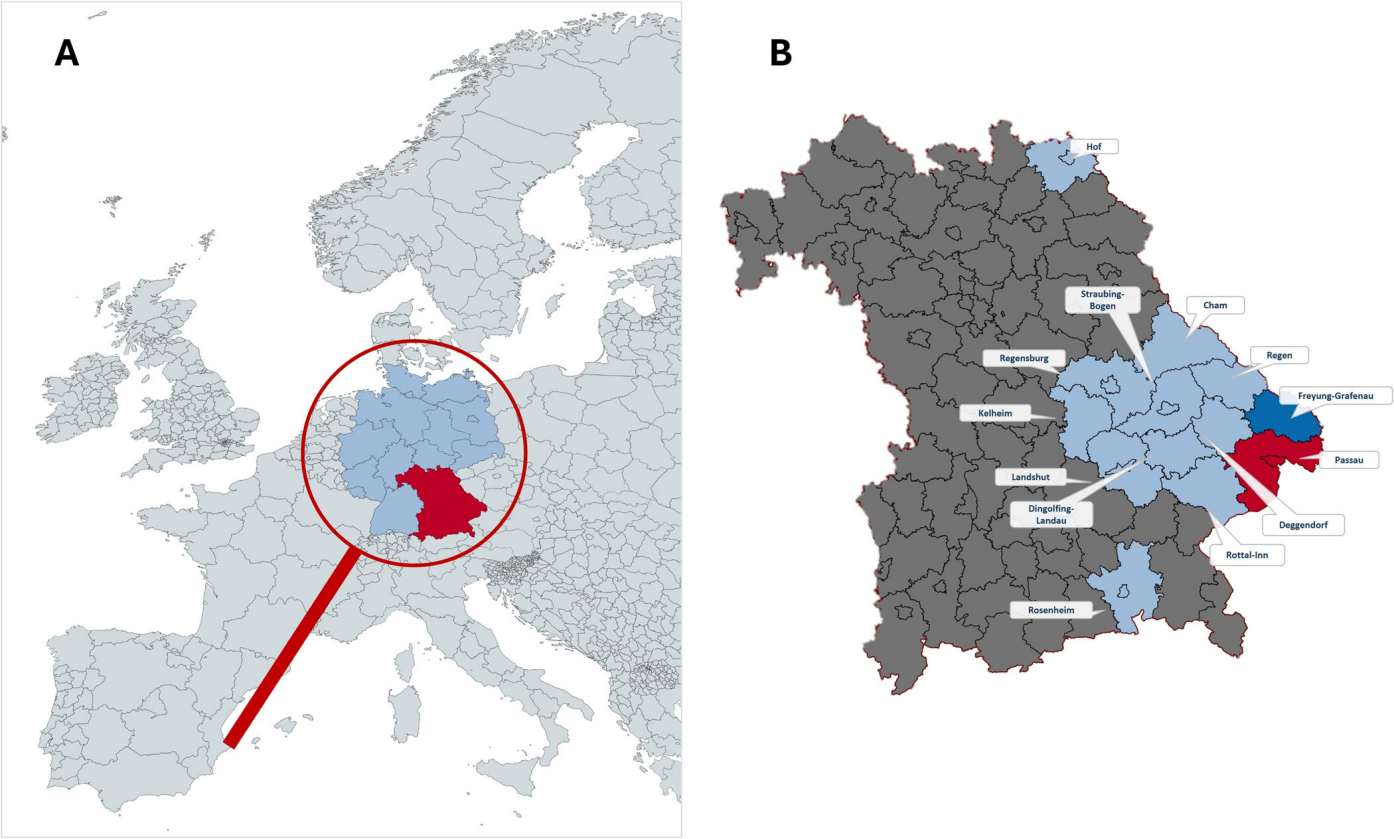
(A) Overview of the study location. (B) Map of Bavaria with the districts analyzed (light blue) and the main regions of Passau (red) and Freyung‐Grafenau (dark blue). *Source:*Overview maps of Europe and Bavaria were created with mapchart.net.

**Figure 3:**
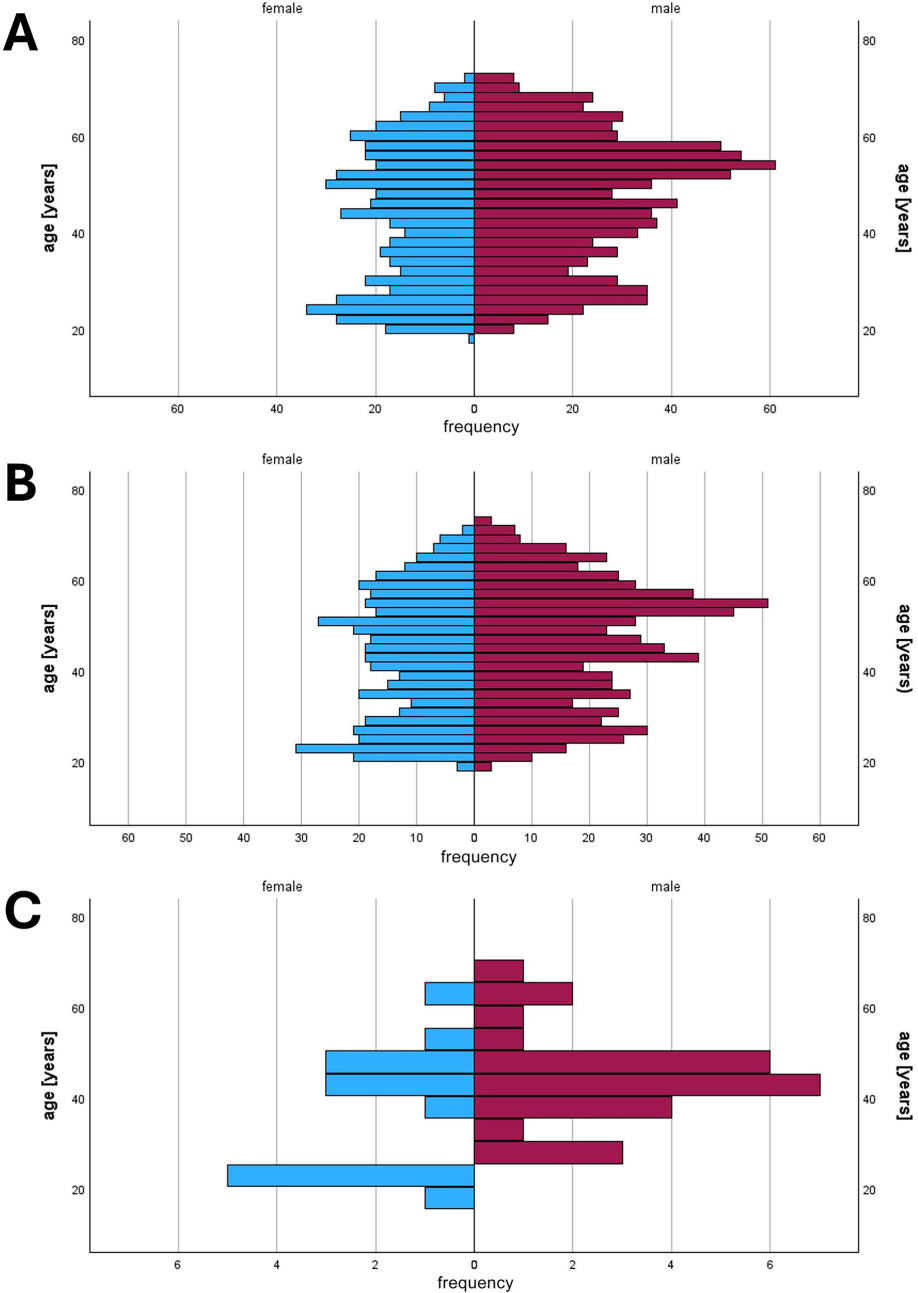
(A) Age and gender distribution in the entire study population, *n* = 1339. (B) Age distribution of the population with active TBEV seroprotection/vaccination (TBEV‐IgG positive and TBEV‐NS1‐IgG negative), *n* = 1094. (C) Age distribution of the population with past TBEV infection (TBEV‐IgG positive and TBEV‐NS1‐IgG positive), *n* = 41.

A total of 1348 human serum samples were collected. Of these, 9 samples were excluded due to an incomplete or incorrect data set. The 1339 samples included in the analysis were from voluntary blood donors aged between 19 and 71 years were analyzed for the study, with an average age of 44.8 years (standard deviation: 13.8 years). The proportion of male donors was 61% (817/1339), the remaining 39% (523/1339) of donors were female. In total, the blood donors came from 14 districts within the administrative region of Lower Bavaria, with the districts of Passau and Freyung‐Grafenau accounting for the vast majority of participants (91.4%) with 983 (73.4%) and 247 (18.4%) blood donors respectively (Figure 2). Further details on the distribution of donors among the individual districts and the age and gender distribution of the donors can be found in Table 1 and Figure 3.

### 3.2 TBEV IgG Antibody Seroprevalence

Of all 1339 sera tested, 1135 (84.8%) were positive for TBEV‐specific IgG antibodies, accordingly, 204 (15.2%) sera tested negative. Further details on the age and gender structure can be found in Figure 3.

### 3.3 TBEV NS1 IgG Seroprevalence

All sera which were reacting in the TBEV IgG ELISA were then analyzed with the NS1‐antibody ELISA as described above to distinguish between antibodies induced by vaccination or infection. Of the 1135 sera analyzed this way, 41 (3.6%) were positive in the NS1‐antibody ELISA, indicating infection‐induced antibodies. The remaining 1094 sera (96.4%) gave a negative result in the NS1‐antibody ELISA, so that a TBEV infection during the last 20 years was unreliable and vaccination‐induced antibodies could be assumed for these sera. In relation to the entire donor group, the determined NS1‐antibody prevalence was 3.1% (41/1339). Further details on the age and gender structure can be found in Figure 3.

### 3.4 NT

To exclude false‐positive reactants due to potential cross‐reactions in the IgG ELISA, all TBEV IgG‐positive sera were additionally analyzed by NT. Here, all 1135 sera were confirmed as positive for TBEV antibodies. Cross‐reactions due to other flavivirus infections or vaccinations therefore can be ruled out for all IgG‐ELISA positive sera, as the NT is considered the gold standard, particularly with regard to its specificity [22]. These results give an overall vaccination rate of 81.2% (1094/1348) for the areas tested.

### 3.5 Incidence

According to current knowledge, an average detectability of NS1‐specific IgG antibodies of ~20 years can be assumed [unpublished data [15]]. In the districts analyzed, the official TBE incidence over the last 20 years was a weighted average of 3.7 per 100 000 inhabitants [8]. We compared this official incidence with the actual incidence that can be calculated from the results of the NS1‐antibody ELISA and thus obtained an actual average TBEV incidence (infection rate)of 180.6 infections per 100 000 inhabitants annually.

### 3.6 Manifestation Index

To calculate the manifestation index, we compared the number of cases reported to the authorities with the number of people actually infected (NS1‐IgG positive). We assumed that the reported cases were symptomatic, as otherwise they would not have been diagnosed. This resulted in an overall manifestation index of 2.1% in the districts analyzed over the last 20 years. Conversely, this means that 97.9% of TBEV infections were asymptomatic or at least went unnoticed by the people affected or by the treating doctors.

### 3.7 Detailed Analysis of the Districts of Passau and Freyung‐Grafenau

With a share of over 90%, the vast majority of the blood donors analyzed came from the two districts of Passau and Freyung‐Grafenau. Therefore, these two districts will be examined in more detail below. The 983 blood donors from Passau were on average 45.4 years old, 62.2% (611/983) were male, and 37.87% (372/983) were female. There were 347 blood donors from Freyung‐Grafenau, who were on average 43.5 years old and 58.3% (144/247) male and 41.7% (103/247) female. The proportion of IgG‐ELISA positive donors was 86.2% (847/983) in Passau and 80.6% (199/247) in Freyung‐Grafenau. The proportion of NS1‐antibody‐positive donors in Passau was 3.4% (33/983) and in Freyung‐Grafenau 2.4% (6/247). According to the officially reported cases, the average annual incidence in the years 2005–2024 was 3.2 (Passau) and 6.6 (Freyung‐Grafenau) per 100 000 inhabitants [13]. In contrast, the actual incidence based on the data from the NS1‐antibody ELISA was 170.0 and 120.0 infections per 100 000 inhabitants, respectively. Taking both incidences into account, the manifestation index for the district of Passau is 1.9%, while that for Freyung‐Grafenau is 5.5%. All values for Passau and Freyung‐Grafenau are summarized in Table 2.

**Table 2:** Comparison of the two main counties included in our analysis—Passau (197 863 inhabitants) and Freyung‐Grafenau (79 603 inhabitants).

	Passau	Freyung‐Grafenau	Total
Sex			
Male	611	144	756
Female	372	103	475
Total *n*	983	247	1230
Mean age in years	45.4	43.5	
Anti‐TBEV‐IgG‐positive, seroprotection/vaccinated	847 (86.2%)	199 (80.6%)	1046 (85%)
Anti‐TBEV‐NS1‐IgG positive, past infection	33 (3.4%)	6 (2.4%)	39 (3.2%)
Reported TBEV incidence per year and 100 000 inhabitants (2005–2024)	3.2	6.6	4.2
Calculated TBEV incidence based on NS1 data	170	120	160
Manifestation index	1.9%	5.5%	2.6%

## 4 Discussion

TBEV is one of the most important tick‐borne viral infections in Europe and Asia. A reliable monitoring and risk assessment by the public health system is of paramount importance. However, since the introduction of TBE vaccines in the 1980s, the lack of differentiation between vaccine and infection antibodies implied that disease surveillance had to rely on rough estimates based on notified human cases. With the new NS1 protein test approach, it is now possible to differentiate between antibodies after vaccination and after infection. This technology now allows to determine the infection risk by determining the seroprevalence and to compare it with the “officially” published prevalence and incidence rates which derive exclusively from reported case numbers.

A total of 1094 out of 1339 donors showed TBEV‐specific IgG antibodies in the screening ELISA while they tested negative in the NS1‐antibody ELISA. Since cross‐reactions and false‐positive ELISA results could be excluded due to the confirmation by NT, a TBE vaccination rate of 81.2% can be assumed. For the two main regions of interest, Passau and Freyung‐Grafenau, the vaccination rate was 82.8% and 78.1%, respectively. These vaccination rates are higher than the rate determined in other studies, where a vaccination coverage of 30%–62% has been assumed for Bavaria [23, 24]. However, these studies are based on estimates, as the direct laboratory‐diagnostic differentiation of vaccine and infection antibodies is not possible without the use of the NS1‐antibody‐based approach—a direct comparison of the figures is therefore difficult. In a recently published study by Euringer and colleagues, the new NS1‐antibody‐based method was used for the first time to determine the prevalence of TBEV in Germany [18]. Euringer and colleagues determined a vaccination rate of 54.9% in a region in Baden‐Württemberg that was also classified as a risk area. In another NS1‐antibody‐based study in western Austria, Siller and colleagues determined a vaccination rate of 77%, while Pilz and colleagues determined a vaccination rate of 81% for Austria nationwide in a survey [23, 25]. As well in a survey‐based study, Pilz and colleagues described a vaccination rate of 62% for Bavaria and 81% for Austria [23]. As the region we sampled around Passau borders directly to Austria, a certain degree of permeability can be assumed, so that the vaccination rate we determined, which is above the Bavarian average, appears plausible. The reasons for these discrepancies in protection rates (i.e. prevalence of neutralizing antibodies) and vaccination rates (rates of documented TBE vaccinations) were discussed in detail earlier [26] and may be mainly due to the counting of incomplete or irregular vaccination schemes which however nevertheless induce neutralizing, i.e. protective antibodies in many instances, but are not counted by official vaccination registers.

It should also be noted that the participants in our study were healthy blood donors, who have a higher awareness of health and prophylaxis. At the same time, this results in a certain restriction of the age distribution of our donors, who cover a range from 19 to 71 years; very young participants or very old participants are not included in our cohort due to specific prerequirement guidelines for blood donors. The use of blood donors could therefore affect the transferability of our results to the population level [27].

Our study also showed a striking difference between the reported TBE incidence and the actual NS1‐antibody incidence as a surrogate for infection. However, our data are in line with other studies from high‐endemic areas in Baden‐Württemberg and Austria: we obtained an NS1‐antibody‐based incidence of 180.6 infections/100 000 inhabitants for the overall group, Euringer and colleagues arrived at the slightly higher value of 283/100 000 (0–1000/100 000) in Baden‐Württemberg and Siller and colleagues were slightly lower in Austria at 136/100 000 (123.3–149.1/100 000) [18, 25]. The resulting average manifestation index of 2.1% is in line with other studies: Euringer and colleagues determined a value of 2% (0%–6%) for Baden‐Württemberg and Siller and colleagues arrived at a value of 1.9% (1.2%–2.6%) in Austria [18, 25]. In Sweden, Albinsson and colleagues found a slightly higher value of 3.1%, but with a wide range of regional differences from 0.4% to 8.7% [28].

When comparing the two main areas of Passau and Freyung‐Grafenau one detail stands out: while the reported incidence and manifestation index for Freyung‐Grafenau is almost twice as high as in Passau (3.5 new cases/100 000 inhabitants and 1.9%, respectively) at 6.6 new cases/100 000 inhabitants and 5.5%, respectively, the NS1‐antibody incidence of 120/100 000 inhabitants is slightly lower than in Passau (170/100 000 inhabitants). The Danube is the natural border between Passau (mainly to the south) and Freyung‐Grafenau (to the north). It is possible that different virus strains with differences in virulence could have established themselves on both sides of this natural barrier. However, this is purely speculative and requires further investigation.

For the calculation of the above‐mentioned values for NS1‐antibody incidence and manifestation index in our study, we assumed that vaccinated patients do not develop sterile immunity but remain susceptible to TBEV and produce NS1‐specific antibodies on contact. The assumed non‐sterile immunity after vaccination is confirmed in a study on vaccinated mice, but it has not been conclusively clarified whether these results can also be transferred to humans [29]. However, personal experience from previous studies and epidemiological data from a recent study in Austria imply that fully vaccinated individuals may no longer produce NS1 antibodies in a subsequent TBEV infection (data not shown [25]). Assuming sterile immunity after TBE vaccination, only the unvaccinated proportion of the donor population should be used to determine the actual (NS1‐antibody‐based) incidence and the manifestation index. This would result in a significantly higher NS1‐antibody‐based incidence of 836.7/100 000 for the entire region or 976.3/100 000 for Passau and 555.6/100 000 for Freyung‐Grafenau. The manifestation index is 2.4 (total), 1.9 (Passau), and 5.5% (Freyung‐Grafenau) if only the unvaccinated proportion is taken into account. These results are also comparable with the results of the other studies: Siller and colleagues in Austria determined an NS1‐based incidence of 610.1/100 000 (557.6–662.5/100 000) and a manifestation index of 1.9% (1.2%–2.9%) for the unvaccinated proportion [25]. In the study by Euringer and colleagues, the parameters among the unvaccinated donors were not yet determined, but their data also indicate a comparable NS1‐antibody‐based incidence of 629.9/100 000 and a manifestation index of 1.65% [18].

It is interesting to compare the actual determined prevalence rate with historical data. In a publication of 1986, Ackermann determined neutralizing antibody prevalence rates in the district of Freyung‐Grafenau of 1.8% and for the district of Passau of 3.9% [30]. These historical data are in line with the prevalence rate found in our study of 3.6% (41/1135 NS1 IgG positive). These data were determined before the introduction of TBE vaccination. Accounting that around 80% of our today's population may be protected from natural TBEV infection, the actual prevalence rate in the nonimmune population has to be calculated with 41/245 (NS1‐IgG negative plus NS1‐IgG positives which at the time of infection must have been negative). This results in a prevalence rate in the nonimmune population of 16.7%, a fivefold higher value as for the whole population. The increase is similar to what was also found for the district of Ortenau [18].

When considering unvaccinated donors in focus, two important points should nevertheless be taken into account: on the one hand, despite an overall efficacy of the available TBE vaccines of over 95%, breakthrough infections can occur, especially in the case of deviations from the vaccination protocol [15]. While studies by Aregay and colleagues suggest that production of NS1‐specific antibodies is significantly impaired in breakthrough infections, this has not been observed so far in our diagnostic ward where now more than 100 breakthrough infections were diagnosed using NS1‐IgG [31]. Nevertheless, we cannot rule out that single vaccine breakthrough infections do not produce NS1‐IgG. This suggests that our estimates are conservative and likely underestimate the true number of infections and incidence rates. However, we do not account a large number of vaccine breakthrough infections in our population as < 3% of our TBEV patients were identified as breakthrough infections [32]. On the other hand, it must be considered that we can currently assume that NS1‐specific antibodies in non‐breakthrough infections can be detected for around 20 years. Again, our calculations will be conservative when assuming that the persistence of NS1‐IgG is shorter than 20 years or lower (and shorter) in breakthrough infections. However, due to the low number of breakthrough infections, this would statistically have only a minor effect on our numbers.

It should also be acknowledged that individual travel history may have influenced the observed incidence rates. However, due to the use of anonymized blood donor sera, it was not possible to control for travel‐related exposure. Despite this limitation, the calculated incidences are consistent with findings from other studies. Notably, the comparison with neighboring Austria—where vaccination coverage is similarly high—supports the validity of our results: the incidence rates reported there were even slightly lower than those observed in our study from Lower Bavaria.

As a TBE vaccination is not usually preceded by testing for pre‐existing specific antibodies, it cannot be ruled out that patients had already formed infection‐induced antibodies before their vaccination. The latter is negligible in Austria, where vaccination campaigns have long been carried out at school age, so that vaccination protection is achieved at a very early age. However, this does not apply to the same extent for Germany, so our data could be distorted by this effect.

One important aspect of this study is also to show the effect of a high vaccination level (> 80%) on the local TBE incidence. The region was selected as the two districts were accounted to have the highest vaccination rates in Southern Germany [24]. Despite the high vaccination rates identified in our data, both the district of Freyung‐Grafenau and the district of Passau have shown an increasing trend in reported TBE cases over the past 10 years [8]. This indicates that even vaccination coverage of 80% or higher, while reducing the overall number of TBE cases, may not be sufficient to halt or reverse the rising trend of TBEV infections in these highly endemic regions. One important aspect in our study is that we tested healthy blood donors. It is known that healthy blood donors form a subpopulation with specific characteristics [27]. This might be of special importance for vaccination studies. However, we assume that the higher the prevalence rate is the less important the healthy donor effect should become. One study showed an overestimation of seroprevalence in SARS‐CoV‐2 in Japan [33]. However, as the main aspect in our study is to compare vaccination‐ versus infection‐induced antibodies, we speculate that both parameters would be overestimated and the proportion of the two parameters will not change dramatically.

In conclusion, our study highlights the significant underestimation of TBEV incidence in high‐risk areas of Lower Bavaria, Germany, when relying only on reported human cases. By applying the NS1‐antibody ELISA‐based approach, we were able to differentiate between vaccine‐ and infection‐induced antibodies, allowing a more accurate assessment of true seroprevalence. Our findings indicate a much higher actual incidence of TBEV infections compared to official data and compared to historical data, emphasizing the need for improved monitoring, reporting, and vaccination efforts. Our results suggest that, despite the high vaccination rate in the region studied, the increasing trend of TBE incidence remains observable—similar to patterns seen in other endemic regions such as Baden‐Württemberg and Austria—although the overall number of infections appears lower in comparison. Future seroprevalence studies utilizing the NS1‐antibody‐based method are essential for a better understanding of TBEV epidemiology and for optimizing public health strategies, including vaccination campaigns and risk communication.

## Author Contributions

Conceptualization, Gerhard Dobler, Silke Martin, and Johannes P. Borde; methodology, Gerhard Dobler and Silke Martin; validation, Philipp Girl, Gerhard Dobler, and Johannes P. Borde; data curation, Philipp Girl, Anne Schindler, and Johannes P. Borde; writing – original draft, Philipp Girl; writing – review and editing, Anne Schindler, Gerhard Dobler, and Johannes P. Borde. All authors have read and agreed to the published version of the manuscript.

## Conflicts of Interest

G.D. and J.P.B. received financial support and lecture fees from Pfizer GmbH Deutschland and Bavarian Nordic GmbH Deutschland.

## Data Availability

Data are available on request from the authors.
